# Supplementary material for: Modes of HIV transmission among young women and their sexual partners in Ukraine
Source: PLoS One. 2024 Jun 26;19(6):e0305072. doi: 10.1371/journal.pone.0305072 (PMC11207155; doi:10.1371/journal.pone.0305072)
Supplement: S1 Table — (DOCX) [file pone.0305072.s001.docx]

S1 Table. Partner recruitment status.

| Number of partners | 0 | 1 | 2 | 3 | 4 | 5 | N (%) >0 |
| --- | --- | --- | --- | --- | --- | --- | --- |
| Number of women who named partners | 7 | 181 | 69 | 28 | 16 | 20 | 314 (98%) |
| Number of women who named 'safe' partners* | 54 | 168 | 52 | 30 | 13 | 4 | 267 (83%) |
| Number of women who agreed to invite partners | 200 | 95 | 16 | 5 | 3 | 2 | 121 (38%) |
| Number of women with partners recruited | 259 | 58 | 4 |  |  |  | 62 (19%) |

* Being ‘safe’ was defined as a negative answer to all three questions: ‘Did this partner ever beat you or cause physical pain by any means?’, ‘Did this partner ever threaten to harm you?’, ‘Did this partner ever force you during sex to do something causing discomfort for you?’
