# Supplementary material for: Modes of HIV transmission among young women and their sexual partners in Ukraine
Source: PLoS One. 2024 Jun 26;19(6):e0305072. doi: 10.1371/journal.pone.0305072 (PMC11207155; doi:10.1371/journal.pone.0305072)
Supplement: S2 Table — (DOCX) [file pone.0305072.s002.docx]

S2 Table. HIV risk factor definitions.

| Injecting drug use | admitting injecting illicit drugs at least once* OR self-reporting the IDU as the most likely way of acquiring HIV OR self-reporting being treated for substance dependence OR self-reporting being treated for overdose |
| --- | --- |
| Homosexual exposure | having one or more male sexual partners* OR having homosexual contact with a man who injected drugs* OR having homosexual contact with an HIV-positive man* OR self-reporting MSM as the most likely way of acquiring HIV OR self-report of rectal sexually transmitted infection (gonorrhea or herpes) |
| Nosocomial exposure | having had blood or blood product transfusion* OR having had organ or tissue transplantation* OR self-reporting being treated for substance dependence* OR self-reporting being treated for overdose* OR being hospitalized for any other reason* OR having had any surgery* OR having been on hemodialysis* OR having had conditions that required frequent injections* OR self-reporting being infected through medical procedures |
| Accidental | Injecting or being injected any substance with a non-sterile needle* OR having been injected by not a medical worker* OR having been involved in practice related to blood, needles or knives (e.g. acupuncture, tattoo, scarring, other practices)* OR self-reporting being infected in a non-occupational accident with skin penetration |
| Sexually transmitted infections | self-report on having gonorrhea OR syphilis OR genital herpes OR other STI at any time before finding out about HIV positive status |

*during 10 years before the first HIV positive test of AGYW.
