# Supplementary material for: Modes of HIV transmission among young women and their sexual partners in Ukraine
Source: PLoS One. 2024 Jun 26;19(6):e0305072. doi: 10.1371/journal.pone.0305072 (PMC11207155; doi:10.1371/journal.pone.0305072)
Supplement: S3 Table — (DOCX) [file pone.0305072.s003.docx]

S3 Table. Comparison of socio-demographic characteristics and HIV risk factors of AGYW, by partner enrollment status.

|  |  |  |  | **Recruited a Partner** | | | |  |  |  |
| --- | --- | --- | --- | --- | --- | --- | --- | --- | --- | --- |
|  |  | **Total** | | **No** | | **Yes** | | **Chi-sq.** | **p-value** |  |
|  |  | **N** | **%** | **N** | **%** | **N** | **%** |  |  |  |
| Total |  | 321 | 100.0 | 259 | 100.0 | 62 | 100.0 |  |  |  |
| Age at the survey | <20 | 12 | 3.7 | 11 | 4.2 | 1 | 1.6 | F | 0.603 |  |
|  | 20-24 | 142 | 44.2 | 116 | 44.8 | 26 | 41.9 |  |  |  |
|  | 25+ | 167 | 52.0 | 132 | 51.0 | 35 | 56.5 |  |  |  |
| Age at HIV registration | <20 | 62 | 19.3 | 48 | 18.5 | 14 | 22.6 | 0.54 | 0.763 |  |
|  | 20-24 | 228 | 71.0 | 186 | 71.8 | 42 | 67.7 |  |  |  |
|  | 25 | 31 | 9.7 | 25 | 9.7 | 6 | 9.7 |  |  |  |
| Education | school | 180 | 56.1 | 144 | 55.6 | 36 | 58.1 | 1.97 | 0.373 |  |
|  | technical | 92 | 28.7 | 72 | 27.8 | 20 | 32.3 |  |  |  |
|  | higher | 49 | 15.3 | 43 | 16.6 | 6 | 9.7 |  |  |  |
| Employment | employed | 166 | 51.7 | 130 | 50.2 | 36 | 58.1 | 4.76 | 0.191 |  |
|  | unemployed | 70 | 21.8 | 57 | 22.0 | 13 | 21.0 |  |  |  |
|  | student | 59 | 18.4 | 53 | 20.5 | 6 | 9.7 |  |  |  |
|  | other | 26 | 8.1 | 19 | 7.3 | 7 | 11.3 |  |  |  |
| Family status | single | 229 | 71.3 | 182 | 70.3 | 47 | 75.8 | F | 0.820 |  |
|  | married | 85 | 26.5 | 71 | 27.4 | 14 | 22.6 |  |  |  |
|  | separated | 7 | 2.2 | 6 | 2.3 | 1 | 1.6 |  |  |  |
| Time from testing to registration | <3 months | 196 | 81.7 | 156 | 80.0 | 40 | 88.9 | F | 0.369 |  |
|  | 3-11.99 months | 25 | 10.4 | 23 | 11.8 | 2 | 4.4 |  |  |  |
|  | 12+ months | 19 | 7.9 | 16 | 8.2 | 3 | 6.7 |  |  |  |
| Time in care | <1 year | 28 | 8.7 | 23 | 8.9 | 5 | 8.1 | 2.26 | 0.521 |  |
|  | 1-1.99 years | 76 | 23.7 | 62 | 23.9 | 14 | 22.6 |  |  |  |
|  | 2-2.99 years | 85 | 26.5 | 64 | 24.7 | 21 | 33.9 |  |  |  |
|  | 3+ years | 132 | 41.1 | 110 | 42.5 | 22 | 35.5 |  |  |  |
| Condom use | never/rarely | 129 | 40.2 | 102 | 39.4 | 27 | 43.5 | 0.60 | 0.743 |  |
|  | 50/50 | 108 | 33.6 | 87 | 33.6 | 21 | 33.9 |  |  |  |
|  | often/always | 84 | 26.2 | 70 | 27.0 | 14 | 22.6 |  |  |  |
| Alcohol/substance use before sex | never | 50 | 15.6 | 43 | 16.6 | 7 | 11.3 | 1.29 | 0.524 |  |
|  | sometimes | 165 | 51.4 | 133 | 51.4 | 32 | 51.6 |  |  |  |
|  | often/always | 106 | 33.0 | 83 | 32.0 | 23 | 37.1 |  |  |  |
| Attended places where others used drugs | no | 295 | 91.9 | 239 | 92.3 | 56 | 90.3 | 0.06 | 0.804 |  |
|  | yes | 26 | 8.1 | 20 | 7.7 | 6 | 9.7 |  |  |  |
| STI history | no | 233 | 72.6 | 193 | 74.5 | 40 | 64.5 | 2.04 | 0.154 |  |
|  | yes | 88 | 27.4 | 66 | 25.5 | 22 | 35.5 |  |  |  |
| Nosocomial exposure | no | 188 | 58.6 | 150 | 57.9 | 38 | 61.3 | 0.12 | 0.733 |  |
|  | yes | 133 | 41.4 | 109 | 42.1 | 24 | 38.7 |  |  |  |
| Accidental exposure | no | 250 | 77.9 | 198 | 76.4 | 52 | 83.9 | 1.20 | 0.274 |  |
|  | yes | 71 | 22.1 | 61 | 23.6 | 10 | 16.1 |  |  |  |
| Sex with male PWID | no | 259 | 80.7 | 211 | 81.5 | 48 | 77.4 | 0.30 | 0.585 |  |
|  | yes | 62 | 19.3 | 48 | 18.5 | 14 | 22.6 |  |  |  |
| Sex with MSM | no | 318 | 99.1 | 257 | 99.2 | 61 | 98.4 | F | 0.476 |  |
|  | yes | 3 | 0.9 | 2 | 0.8 | 1 | 1.6 |  |  |  |
| Sex with male PLWH | no | 133 | 41.4 | 119 | 45.9 | 14 | 22.6 | 10.31 | **0.001** |  |
|  | yes | 188 | 58.6 | 140 | 54.1 | 48 | 77.4 |  |  |  |
| Sex with male sex worker | no | 314 | 97.8 | 254 | 98.1 | 60 | 96.8 | F | 0.624 |  |
|  | yes | 7 | 2.2 | 5 | 1.9 | 2 | 3.2 |  |  |  |
| Selling sex for money | no | 311 | 96.9 | 256 | 98.8 | 55 | 88.7 | F | **0.001** |  |
|  | yes | 10 | 3.1 | 3 | 1.2 | 7 | 11.3 |  |  |  |
| History of IPV with named partners | no | 280 | 87.2 | 228 | 88.0 | 52 | 83.9 | 0.45 | 0.503 |  |
|  | yes | 41 | 12.8 | 31 | 12.0 | 10 | 16.1 |  |  |  |
| SMoT, survey-based mode of HIV transmission; IDU, injecting drug use; AGYW, adolescent girls and young women; STI, sexually transmitted infection; PWID, people who inject drugs; MSM, men who have sex with men; PLWH, people living with HIV, IPV, intimate partner violence. F denotes that Fischer’s exact test was used instead of chi-square test. | | | | | | | | | | |
