# Supplementary material for: Modes of HIV transmission among young women and their sexual partners in Ukraine
Source: PLoS One. 2024 Jun 26;19(6):e0305072. doi: 10.1371/journal.pone.0305072 (PMC11207155; doi:10.1371/journal.pone.0305072)
Supplement: Attachment — Submitted filename: Review_PWID and AGYW in Ukraine.docx [file pone.0305072.s006.docx]

The paper describes a study investigating modes of HIV transmission in adolescent girls and young women in Ukraine and their links to PWID. I believe that this study addresses important questions, especially for an Eastern European country with the high prevalence of injecting drug use and HIV. The study utilizes both a survey and serological confirmatory testing, along with obtaining information from the national HIV database. The study participants were enrolled from nine different regions of Ukraine, which is a great strength considering the size of the country and the known disparity in healthcare, HIV prevalence, and economy between the regions.

I believe this paper will be of interest to readers, and I hope the comments below can improve it and eliminate the concerns that might arise.

I think that this paper could be substantially improved by shortening the Introduction section and focusing only on the most relevant findings of the previous studies.

It was not fully clear for me as a reader that the research question is the prevalence of HCV in partners of the AGYW enrolled in the study. A substantial emphasis is put on AGYW, the section on their partners in Result is the second one, and I only found that the main question was the prevalence of HCV in partners, from Supplementary 2. The authors might consider reshaping the paper by starting the Results section from the merged Table 1 including both AGYW and their partners, then going by the order of their hypotheses as they were listed in Supplementary 3.

I would suggest to also emphasize the main research question more in Discussion. I believe there is room for comparison also with non-Eastern European studies, especially given that the authors want to stress the role of stigma in IDU underreporting and the higher barriers to accessing care in Ukraine.

I would advise the authors to check the paper against the People First Chapter terminology guidance, specifically avoiding such words as “HIV-infected” or “having HIV seropositive status” which can be replaced by “living with HIV” (<https://peoplefirstcharter.org/#:~:text=The%20People%20First%20Charter%20was,language%20perpetuates%20stigma%20%26%20marginalises%20people>)

I believe some check of the references is needed; in a couple of places, the information in the paper does not match the data in the references.

I also believe this paper could be slightly improved also in terms of the language and grammar; currently, there are some places where the meaning of the text is unclear, and where the language check will improve the readability.

**Title and keywords:**

You could consider not using the keyword “surveillance”, given that your paper does not describe systematic and regular data collection, but a one-time survey and HCV testing data collection. Maybe “women with HIV” or “Ukraine” could be considered more relevant keywords.

**Abstract:**

The text of this paper might benefit from some grammatical corrections, for example in ”***We explored how PWID sexual partners may be a source of heterosexual HIV transmission***” I would suggest using “whether”/”if” instead of “how”, and the sentence “***Never or rarely used condoms 51% of Partners and 23% were not linked to HIV care***” will sound clearer the following way: “*Fifty-one percent of partners never or rarely used condoms, and 23% were not linked to HIV care*”. In some places, the word “partners” starts with a capital letter, which should be either corrected in a uniform way, or defined if “Partners” with a capital letter is a separate category that includes, e.g. only HIV positive men.

***”Reports indicate over 11 million 57 PWID globally, of whom 1.4 million have an HIV-seropositive diagnosis”*** . This data comes from the UNODC World Drug Report 2020 (https://wdr.unodc.org/wdr2020/field/WDR20_BOOKLET_1.pdf), and the WHO webpage you cited, is referencing this source. Please consider replacing “have an HIV-seropositive diagnosis” with “living with HIV”, in line with the wording in the UNODC report and on the WHO website.

I would also advise against the wording “***PWID are responsible for XX% of HIV infections***”, and would rather suggest using “*account for*”, in line with the wording in the WHO Global health sector strategy.

Please check the references: currently, ref. 8 and 9 include the link on the same webpage, which, for some reason, also does not open on my PC. (I apologise in advance if this is a technical issue on my part).

Reference 10 does not investigate transmission modes in new HIV diagnoses – could there be a mistake in the reference numbering?

Reference 11, used for the sentence “***Current evidence suggests that PWID and their sexual partners are responsible for up to 65% of all new HIV infections worldwide***”, sounds as “*In 2020, key populations and their sexual partners accounted for 65% of the people acquiring HIV globally*”, where key populations are defined as “men who have sex with men, people who inject drugs, sex workers, transgender people, and people in prisons and other closed settings”.

Starting from Line 66 and till Line 108, I would suggest shortening the literature review part. The purpose of the Introduction section is to outline the background in a concise way, focusing on the points most relevant to understand the context of your study. I believe a substantial part of the details can be left out or moved to Discussion if they are important for comparison with your study results. One of the possible rewording options to keep the section concise and to the point, could be the following (an example for lines 66-87):

“*Additionally, previous studies found that PWID often engage in risky sexual practices, such as having unprotected sex or having multiple sexual partners, who are often non-PWID (Tun et al., Niccolai et al.)*”.

Starting from Line 113, you use an abbreviation “IDU”: please spell out the abbreviation the first time when you used it.

Line 109-110: please check the reference, which is now listed as “***UNAIDS. Global AIDS Monitoring 2019: Ukraine 2020***”. The estimated number of PWH in Ukraine was around 240,000 in 2018, and increased to 250,000 according to the UNAIDS Global AIDS Monitoring 2019 report <https://www.unaids.org/sites/default/files/country/documents/UKR_2020_countryreport.pdf>

Please also consider rewording Lines 110-111, as they now read as as “of all PWH who inject drugs, 20% live in Ukraine” and not as “of all PWH in Ukraine, 20% are PWID”.

Lines 111-113: the reference is the report from 2016, therefore you could only say that heterosexual mode of transmission was the main one from 2008 to 2016 if you do not have later data. Perhaps later reports are available? For example, Information Bulletin #51 from 2020 is describing 2019 data and perhaps could be cited instead.

Lines 117-118: the UNAIDS report, although published in 2019, presents the estimates for 2018; also, it seems that this is not the number of AGYW but the number of all girls and women living with HIV older than 15 years (<https://www.unaids.org/sites/default/files/media_asset/unaids-data-2018_en.pdf>) .

Lines 117-121: “***According to the UNAIDS report [31], in 2019, approximately 110 000 AGYW lived with HIV in Ukraine. Risk factors include early sexual debut, unprotected sex, and having sexual partners who engage in high-risk behaviours such as drug use, underscoring prominent gender differences in contracting HIV [32]***”. Reference 32 is a study performed exclusively in PWID, whereas your paper only presents results for women with heterosexual HIV transmission mode. If you want to describe risk factors in the target population, I would advise to look for a reference in the group that is closer to your study population; otherwise, you might want to delete this sentence, given that risk factors are not related to your main research question.

Lines 122-124 refer to the citations dated 2012 and 2009; given that over ten years have passed since then, I believe another reference should be found to confirm that heterosexual transmission among women in Ukraine ***remains*** primarily linked to PWID partners.

Lines 124-126: I believe the wording should be changed for clarity (“*challenges to accessing preventive measures*”, but “*barriers to HIV disclosure*”)

Lines 126-127: “The growing intersecting epidemics of HIV and drug use pose intrinsic concerns about the AGYW's autonomy to seek preventative HIV 128 care in Ukraine” – As per the official HIV report of the PHC of the MoH of Ukraine (HIV infection in Ukraine, newsletter #51) the proportion of parenteral HIV transmission decreased from 42% in 2010 to 26% in 2019, therefore I am not sure you could say it’s growing

Line 131-132: “***AGYW non-PWID sexual partners of PWID***” – this is a long line of abbreviations which does not read very clear. If your study includes data on AGYW who are non-PWID, but not all of them are sexual partners of PWID, perhaps the phrase could be shortened to “AGYW who are not PWID” or “AGYW not using drugs”.

Line 136: the definition of the “bridge populations” was not provided before; “potential bridging partnerships” were mentioned in the context of the Russian study, but I would advise to specify what was the definition of the bridge population used in your study; or call it “potential bridging partnerships” if you used the same definition as the Russian study; or shorten to “This study aimed to investigate the possible link between PWID and AGYW who were registered in HIV care with the heterosexual mode of HIV transmission across nine regional HIV clinics in Ukraine”.

**Methods:**

I believe that after shortening the Introduction section by making it a background outline instead of a literature review, you will have more spare word count and will be able to include the most important points on statistical analysis in the text of the Methods section. This would include statistical tests, whereas the definitions of the logical formulas can be kept as a supplementary.

For all tables in this section, I would suggest adding lines for rows and columns, to make it easier to identify which variable is reflected by each proportion.

Lines 146-153: In the supplementary document you mentioned that participants were enrolled using random selection from the national database; I suggest including this in Methods, as it is most important information about the study population.

Lines 154-157: It might be of interest if the number of enrolled participants was proportionally distributed across nine clinics or four geographical regions?

Line 165: “***Specifically, those females who tested 166 HCV positive or reported IDU or reported no sexual relationships with men prior to their HIV diagnosis were defined as new HIV cases with likely IDU mode of HIV transmission***” – “reported” is reported twice, could be changed to “***those females who tested 166 HCV positive or reported either IDU or no sexual relationships with men prior to their HIV diagnosis were defined as new HIV cases with likely IDU mode of HIV transmission”.***

Line 168: ***Those AGYW who tested HCV negative and reported no IDU and reported having sexual relationships with men prior to HIV diagnosis were defined as new HIV cases with likely heterosexual mode of HIV transmission*** – similar to the above.

Line 173: “**Partners were HIV-positive men or men with unknown HIV status with whom the AGYW had sexual relationships prior to their HIV diagnosis and indicated them as a potential source of HIV infection**” – It is not clear for me whether a woman referred up to 5 most recent partners before the HIV diagnosis, or a woman only referred those partners whom she saw as a potential person she acquired HIV from? (i.e. to which extent it was based on her opinion on partners)

Line 185: “***In addition, each potential Partner indicated by AGYW was screened for intimate partner violence (IPV)”*** – I do not see IPV as an exclusion criterion in the supplementary file describing study enrolment. It is also not clear for me why did you decide using IPV event as exclusion criterion; could you please elaborate on that?

**Results:**

A general limitation reg. the analysis is that in this population, even using a few variables, you deal with confounding. Most obvious example: age is correlated with educational level. If any adjustment is not possible in your study sample, I would advise mentioning this point as a study limitation.

I think it might be of interest to indirectly look at the ‘missing’ group of the partners who did not participate in the study. Was there any age, educational level, or risk behavior difference between the women whose partners agreed to participate, and those whose did not? Was any difference in IPV between these two groups of women? The missing group is likely not missing at random, as one would hypothesize that a person who inject drugs might be more likely to refuse to participate.

Could you please explain why the age group were defined using the cutoff of 26 years? At inclusion, you used a definition of 15-25 years, and five years seem also the more logical time interval for an ‘arbitrary’ age group definition; why 26 years was used at the study enrolment?

Line 221-224: “***236 (73%) AGYW indicated at least one sexual partner with negative IPV screening results, and 94 (40%) agreed to refer their partners to the study site. 41 (13%) out of all AGYW reported IPV history.***” – If 73% reported at least one partner with negative IPV screening result, and 13% reported IPV history, even assuming that no IPV history was ever reported in the 73% group, there are still 10% with neither positive nor negative IPV, could you please elaborate on this?

For the question on family status, the answers are “single/separated” or “married”. Was cohabitance without marriage reported as “single”?

You could consider excluding the variable “IDU exposure” and “Heterosexual exposure” from Table 1, given that this was an exclusion and inclusion criterion, respectively, therefore by definition you would not have anyone with IDU exposure in your population, and all will be heterosexual.

“Time in HIV” care groups look overlapping, whereas the total proportion shows no overlap in participants. Was Year 2 in care included in the “1-2 years” or in the “2-3 years” interval? Same question for the Year 3.

The numbers of participants for the three of the four years of registration in care is precisely the same: 85. Was this planned at the selection stage (i.e. you planned to select the same number of participants for each of the study years)? If so, this should be specified in Methods or in Supplementary 2.

Line 228: “***Sex with PWID before HIV diagnosis was reported in 19% of females, and rare or never condom use was reported in 40% of females***” – consider replacing “females” with “women”

Table 2: I suggest deleting “or drugs” and keeping only “Exchange money for sex”, for the same reason I do not recommend keeping “IDU exposure” in Table 1.

Lines 227-231: here in the text, you report the same proportions that are listed in the table. You may consider shortening the text, to minimize duplicate data. I would suggest removing the sentence on sex with MSM and commercial partners.

Lines 234-236: “***AGYW who reported having IDU sexual partner (IP) had a significantly higher frequency of high-risk heterosexual exposure than AGYW reporting having a non-IDU partner (NIP) (100% vs 59%, p<.0001****)”.* Here, the outcome of interest is used for the group definition, and in this case, there is no variance in one of the groups (IDU partners) and your p will be low even if only one of the participants in the non-IDU partner group does not have a high-risk exposure. You might consider different methodology to compare to a ceiling, or easier, you could simply say “*among AGYW reporting having a non-IDU partner, the frequency of high-risk heterosexual exposure was 59% (95%CI XX-XX)”.*

I believe the sample size is also too small to assess the difference between groups for exchanging money for sex; sex with MSM; and sex with commercial partner, therefore p-value is meaningless, and I would suggest excluding the sentence describing p-values in these groups.

Lines 236-238: “***In addition, AGYW with IP were more likely to have STI exposure and sex with PLWH than AGYW with NIP (40% vs 24%, p=.011 and 74% vs 55%, p=.005 respectively)”.*** How was “sex with PLWH” defined? Could the woman answer based on her suspicion, or should there be any confirmation of the partner’s HIV status? Additionally, please spell out “PLWH” – it seems for me as it was not spelled before.

Unfortunately, I could not properly look at Table 3, since a part of it was not visible in the portrait format. Please include this table in landscape format. Additionally, you could shorten the text above the table leaving only the most important findings, to avoid duplicating a large amount of data that is already in the table.

Line 241: ”***Rare/never condom use was numerically higher than AGYW with IP***” - a typo: “*was higher IN AGYW with IP*”

Lines 257-258: “***and 71% reported consuming alcohol and drugs before sex always or frequently***” – should it rather be “*alcohol or drugs*”?

In the analysis in partners, some subgroups are very small for the p-value to be able to estimate the difference.

In table 5, 0% of partners reported exchanging money or drugs for sex but 33% and 19% reported having sex with a commercial partner. Could you please elaborate on that? Does it mean that the partners in fact replied to the question of “exchanging sex for money or drugs”, i.e. selling, but not buying sex?

**Discussion:**

Lines 290-291: “***Our results indicate that AGYW with IDU sexual partners are at higher odds for high-risk heterosexual exposure than non-IDU partners***” – As mentioned in the comments to the Results section, having an IDU sexual partner is one of the definitions of the high-risk behavior (something like if you say “people who were born in Ukraine are more likely to have ever lived in Ukraine than those who were born in another country”).

Lines 291-293: “***In addition, having an IDU sex partner was associated with a higher probability of STI exposure and being more likely to have sex with PLWH***”. – I believe this can be strengthened by the check in the group of women whose partners participated in the study, because in this case, you have a confirmation of the HIV status from the partner or HIV clinic. Have you considered looking into it?

Lines 293-295: “***Similarly, in the study conducted by Deryabina et al. [47] in similar Post-Soviet contextual settings, up to 70% of women reported risky sexual behaviour with their PWID partners***” – The paper does not assess any of the high-risk definitions used in your analysis apart from the sex with PWID (which was an inclusion criteria). For clarity of the comparison, I would advise specifying which parameter was reported by up to 70% women in this study.

Lines 319 – 321: “***Furthermore, the proportion of Partners who in our survey self-reported IDU at the time prior to AGYW HIV diagnosis was strikingly higher than those who have been registered in HIV clinics with IDU mode of HIV transmission***”. This does not seem in line with the Results section:

Lines 262-263: ***More than half (53%) of all Partners were registered in HIV clinic with a heterosexual mode of transmission, and 24% were registered as IDU***.

Lines 265-266: ***During the survey, 19 (29%) of Partners reported IDU as their mode of HIV infection***.

The difference between the 24% registered as IDU and 29% who reported IDU transmission during the survey, does not appear to be very striking.

Line 343: “***more lifetime sexual partners and STDs”*** – please spell out “STD” or use STI if this is the same

Lines 360-371: I suggest shortening or even deleting this part, considering that HIV disclosure is not investigated in this paper, and its importance is mentioned in the other parts of the manuscript.

Limitations: I would suggest mentioning that, along with the low number of enrolled partners, it is also possible that those who did not respond to the referral, were not missing at random.

**Supplementary 1:**

Could you please explain why it has been decided to link one partner to one woman only? Potentially, there is no obstacle for one man to infect multiple women.
