# Supplementary material for: Modes of HIV transmission among young women and their sexual partners in Ukraine
Source: PLoS One. 2024 Jun 26;19(6):e0305072. doi: 10.1371/journal.pone.0305072 (PMC11207155; doi:10.1371/journal.pone.0305072)
Supplement: Attachment — Submitted filename: Response to Reviewers.docx [file pone.0305072.s007.docx]

The paper describes a study investigating modes of HIV transmission in adolescent girls and young women in Ukraine and their links to PWID. I believe that this study addresses important questions, especially for an Eastern European country with the high prevalence of injecting drug use and HIV. The study utilizes both a survey and serological confirmatory testing, along with obtaining information from the national HIV database. The study participants were enrolled from nine different regions of Ukraine, which is a great strength considering the size of the country and the known disparity in healthcare, HIV prevalence, and economy between the regions.

I believe this paper will be of interest to readers, and I hope the comments below can improve it and eliminate the concerns that might arise.

RESPONSE

We thank the Reviewer for a thoughtful and detailed review and, agreeing with the comments, have revised the manuscript significantly.

I think that this paper could be substantially improved by shortening the Introduction section and focusing only on the most relevant findings of the previous studies.

RESPONSE

We agree, and the Introduction was substantially shortened to focus on the main research question – heterosexual HIV transmission between key and bridge populations.

It was not fully clear for me as a reader that the research question is the prevalence of HCV in partners of the AGYW enrolled in the study. A substantial emphasis is put on AGYW, the section on their partners in Result is the second one, and I only found that the main question was the prevalence of HCV in partners, from Supplementary 2. The authors might consider reshaping the paper by starting the Results section from the merged Table 1 including both AGYW and their partners, then going by the order of their hypotheses as they were listed in Supplementary 3.

We apologize for the confusion. The analytic approach and the manuscript were substantially revised to highlight the main research question - heterosexual HIV transmission between key and bridge populations. The primary study population is AGYW, and the proportion of cases that can be linked to PWID is the main study indicator. The new table 1 describes the characteristics of the sampled primary population, both in terms of socio-demographic characteristics and HIV risks. We recruited their partners to ascertain their mode of transmission (which may be unknown to the referring women and also misclassified in the registration system). The characteristics of Partners are presented in Table 2, including their survey-based mode of transmission. Then, we compare AGYW whose Partners are likely PWID, to those who are not likely PWID (Table 3).

We hope that this approach is more understandable and highlights the main study questions and findings.

I would suggest to also emphasize the main research question more in Discussion. I believe there is room for comparison also with non-Eastern European studies, especially given that the authors want to stress the role of stigma in IDU underreporting and the higher barriers to accessing care in Ukraine.

Discussion was revised thoroughly to emphasize the main research question. We also reference several global reviews and studies from Central Asia about stigma and IPV.

I would advise the authors to check the paper against the People First Chapter terminology guidance, specifically avoiding such words as “HIV-infected” or “having HIV seropositive status” which can be replaced by “living with HIV” (<https://peoplefirstcharter.org/#:~:text=The%20People%20First%20Charter%20was,language%20perpetuates%20stigma%20%26%20marginalises%20people>)

Thank you for this suggestion, we fully support this guidance. We revised the language to avoid the word ‘infected’, however when we refer to the mode of HIV acquisition (e.g. ‘infected through IDU’) we thought it was appropriate. We would be happy to revise further if there are better alternatives in this context.

I believe some check of the references is needed; in a couple of places, the information in the paper does not match the data in the references.

Thank you, we agree that some references were misplaced. We have checked all references and believe they now match the points we make.

I also believe this paper could be slightly improved also in terms of the language and grammar; currently, there are some places where the meaning of the text is unclear, and where the language check will improve the readability.

We have revised the language in many places and checked grammar using advanced tools, and hope it is now more correct and clearer.

**Title and keywords:**

You could consider not using the keyword “surveillance”, given that your paper does not describe systematic and regular data collection, but a one-time survey and HCV testing data collection. Maybe “women with HIV” or “Ukraine” could be considered more relevant keywords.

Thank you, we agree, the keyword is removed.

**Abstract:**

The text of this paper might benefit from some grammatical corrections, for example in ”***We explored how PWID sexual partners may be a source of heterosexual HIV transmission***” I would suggest using “whether”/”if” instead of “how”, and the sentence “***Never or rarely used condoms 51% of Partners and 23% were not linked to HIV care***” will sound clearer the following way: “*Fifty-one percent of partners never or rarely used condoms, and 23% were not linked to HIV care*”.

Thank you, we have revised the language.

In some places, the word “partners” starts with a capital letter, which should be either corrected in a uniform way, or defined if “Partners” with a capital letter is a separate category that includes, e.g. only HIV positive men.

We apologize for the confusion. We use ‘partners’ when we talk about people who are sexual or injecting contacts in general and outside of the study context. To refer to the specific study population and the recruited sample, we use the capitalized term ‘Partners’. We have added clarification in parentheses in both Abstract and Methods.

***”Reports indicate over 11 million 57 PWID globally, of whom 1.4 million have an HIV-seropositive diagnosis”*** . This data comes from the UNODC World Drug Report 2020 (https://wdr.unodc.org/wdr2020/field/WDR20_BOOKLET_1.pdf), and the WHO webpage you cited, is referencing this source. Please consider replacing “have an HIV-seropositive diagnosis” with “living with HIV”, in line with the wording in the UNODC report and on the WHO website.

This part was now removed to shorten Introduction.

I would also advise against the wording “***PWID are responsible for XX% of HIV infections***”, and would rather suggest using “*account for*”, in line with the wording in the WHO Global health sector strategy.

Thank you. Corrected.

Please check the references: currently, ref. 8 and 9 include the link on the same webpage, which, for some reason, also does not open on my PC. (I apologise in advance if this is a technical issue on my part).

Reference 10 does not investigate transmission modes in new HIV diagnoses – could there be a mistake in the reference numbering?

Introduction has been substantially shortened, and the remaining references were checked.

Reference 11, used for the sentence “***Current evidence suggests that PWID and their sexual partners are responsible for up to 65% of all new HIV infections worldwide***”, sounds as “*In 2020, key populations and their sexual partners accounted for 65% of the people acquiring HIV globally*”, where key populations are defined as “men who have sex with men, people who inject drugs, sex workers, transgender people, and people in prisons and other closed settings”.

Thank you. Corrected to “Current evidence suggests that HIV key populations, including PWID, and their sexual partners account for up to 65% of all new HIV infections worldwide”

Starting from Line 66 and till Line 108, I would suggest shortening the literature review part. The purpose of the Introduction section is to outline the background in a concise way, focusing on the points most relevant to understand the context of your study. I believe a substantial part of the details can be left out or moved to Discussion if they are important for comparison with your study results. One of the possible rewording options to keep the section concise and to the point, could be the following (an example for lines 66-87):

“*Additionally, previous studies found that PWID often engage in risky sexual practices, such as having unprotected sex or having multiple sexual partners, who are often non-PWID (Tun et al., Niccolai et al.)*”.

Thank you, we agree, Introduction has been substantially shortened, this sentence is now in paragraph 1.

Starting from Line 113, you use an abbreviation “IDU”: please spell out the abbreviation the first time when you used it.

We apologize for the confusion, in the previous version terms ‘IDU’ and ‘PWID’ were sometimes used interchangeably. Now we have corrected this to make it clear that ‘IDU’ refers to injecting drug use, i.e. behavior or mode of transmission, whereas ‘PWID’ refers to people who inject drugs.

Line 109-110: please check the reference, which is now listed as “***UNAIDS. Global AIDS Monitoring 2019: Ukraine 2020***”. The estimated number of PWH in Ukraine was around 240,000 in 2018, and increased to 250,000 according to the UNAIDS Global AIDS Monitoring 2019 report <https://www.unaids.org/sites/default/files/country/documents/UKR_2020_countryreport.pdf>

The source for the PLWH estimate in Ukraine is Spectrum modeling, and the latest report provides re-calculated figure which is 240,000 for 2020:

<https://phc.org.ua/sites/default/files/users/user90/Natsionalna_otsinka_sytuatsyi_z_VIL_SNIDu_v_Ukraini_na_pochatok_2022.pdf>.

We now cite this latest publication.

Please also consider rewording Lines 110-111, as they now read as as “of all PWH who inject drugs, 20% live in Ukraine” and not as “of all PWH in Ukraine, 20% are PWID”.

This paragraph has been revised, to start from the general PLWH estimate, and then explaining the proportion of PWID among them.

Lines 111-113: the reference is the report from 2016, therefore you could only say that heterosexual mode of transmission was the main one from 2008 to 2016 if you do not have later data. Perhaps later reports are available? For example, Information Bulletin #51 from 2020 is describing 2019 data and perhaps could be cited instead.

This paragraph has been revised, hopefully providing an accurate explanation of the trends in modes of transmission in Ukraine.

Lines 117-118: the UNAIDS report, although published in 2019, presents the estimates for 2018; also, it seems that this is not the number of AGYW but the number of all girls and women living with HIV older than 15 years (<https://www.unaids.org/sites/default/files/media_asset/unaids-data-2018_en.pdf>) .

Thank you, indeed that figure represented all women living with HIV in Ukraine. We have now replaced it with data from the Spectrum report.

Lines 117-121: “***According to the UNAIDS report [31], in 2019, approximately 110 000 AGYW lived with HIV in Ukraine. Risk factors include early sexual debut, unprotected sex, and having sexual partners who engage in high-risk behaviours such as drug use, underscoring prominent gender differences in contracting HIV [32]***”. Reference 32 is a study performed exclusively in PWID, whereas your paper only presents results for women with heterosexual HIV transmission mode. If you want to describe risk factors in the target population, I would advise to look for a reference in the group that is closer to your study population; otherwise, you might want to delete this sentence, given that risk factors are not related to your main research question.

Thank you for noting this, we have now added two references specific for AGYW, one of them is from Ukraine.

Lines 122-124 refer to the citations dated 2012 and 2009; given that over ten years have passed since then, I believe another reference should be found to confirm that heterosexual transmission among women in Ukraine ***remains*** primarily linked to PWID partners.

This is a very good point. We have now moved this sentence to the paragraph describing the trends in modes of transmission, to put it into historical perspective. In fact, there were no recent studies that would estimate the extent of heterosexual transmission between key and bridge populations, but it was experts’ belief that it is largely connected, which is also mentioned in the UNAIDS report (reference added). This is exactly why our study commenced – to confirm that hypothesis. We hope that in such a historical perspective these citations are appropriate.

Lines 124-126: I believe the wording should be changed for clarity (“*challenges to accessing preventive measures*”, but “*barriers to HIV disclosure*”)

Thank you, corrected.

Lines 126-127: “The growing intersecting epidemics of HIV and drug use pose intrinsic concerns about the AGYW's autonomy to seek preventative HIV 128 care in Ukraine” – As per the official HIV report of the PHC of the MoH of Ukraine (HIV infection in Ukraine, newsletter #51) the proportion of parenteral HIV transmission decreased from 42% in 2010 to 26% in 2019, therefore I am not sure you could say it’s growing

The sentence was deleted.

Line 131-132: “***AGYW non-PWID sexual partners of PWID***” – this is a long line of abbreviations which does not read very clear. If your study includes data on AGYW who are non-PWID, but not all of them are sexual partners of PWID, perhaps the phrase could be shortened to “AGYW who are not PWID” or “AGYW not using drugs”.

Agreed, the sentence was revised as ‘..HIV transmission from PWID to AGYW who do not inject drugs’.

Line 136: the definition of the “bridge populations” was not provided before; “potential bridging partnerships” were mentioned in the context of the Russian study, but I would advise to specify what was the definition of the bridge population used in your study; or call it “potential bridging partnerships” if you used the same definition as the Russian study; or shorten to “This study aimed to investigate the possible link between PWID and AGYW who were registered in HIV care with the heterosexual mode of HIV transmission across nine regional HIV clinics in Ukraine”.

The definition of bridge populations from the National HIV Program is provided now in paragraph 2, it is also applicable to our study.

**Methods:**

I believe that after shortening the Introduction section by making it a background outline instead of a literature review, you will have more spare word count and will be able to include the most important points on statistical analysis in the text of the Methods section. This would include statistical tests, whereas the definitions of the logical formulas can be kept as a supplementary.

Thank you for this suggestion, we totally agree, and have revised the section completely. We believe that all the important information about selection and recruitment is now included in the main text. All supplements about methods are now removed, except for the table with risk factor definitions.

For all tables in this section, I would suggest adding lines for rows and columns, to make it easier to identify which variable is reflected by each proportion.

We understand this comment refers to the tables in Results. We have formatted the new tables using horizontal borders, separating all variables. But we believe that the general convention for journal publications is not to use vertical borders. We can add them if Reviewers and Editors will suggest that it would improve clarity.

Lines 146-153: In the supplementary document you mentioned that participants were enrolled using random selection from the national database; I suggest including this in Methods, as it is most important information about the study population.

Agreed, this information was added to the text.

Lines 154-157: It might be of interest if the number of enrolled participants was proportionally distributed across nine clinics or four geographical regions?

The target sample size was indeed distributed proportionally to the total number of HIV cases registered in 2016-2019. However, during the COVID pandemic the participating clinics were affected to a varying extent, therefore the final sample was not strictly proportional.

Line 165: “***Specifically, those females who tested 166 HCV positive or reported IDU or reported no sexual relationships with men prior to their HIV diagnosis were defined as new HIV cases with likely IDU mode of HIV transmission***” – “reported” is reported twice, could be changed to “***those females who tested 166 HCV positive or reported either IDU or no sexual relationships with men prior to their HIV diagnosis were defined as new HIV cases with likely IDU mode of HIV transmission”.***

This sentence was revised as: Women who reported IDU or were HCV-positive were excluded from the study, as they were considered to have acquired HIV via parenteral mode.

Line 168: ***Those AGYW who tested HCV negative and reported no IDU and reported having sexual relationships with men prior to HIV diagnosis were defined as new HIV cases with likely heterosexual mode of HIV transmission*** – similar to the above.

This sentence was deleted.

Line 173: “**Partners were HIV-positive men or men with unknown HIV status with whom the AGYW had sexual relationships prior to their HIV diagnosis and indicated them as a potential source of HIV infection**” – It is not clear for me whether a woman referred up to 5 most recent partners before the HIV diagnosis, or a woman only referred those partners whom she saw as a potential person she acquired HIV from? (i.e. to which extent it was based on her opinion on partners)

We asked specifically about sexual partners who could be a source of HIV, i.e. those with whom they had contacts before the diagnosis. The sentence was revised: AGYW were requested to identify up to five men with whom they had sexual contacts before their HIV diagnosis and who could be a potential source of HIV infection (‘Partners’).

Line 185: “***In addition, each potential Partner indicated by AGYW was screened for intimate partner violence (IPV)”*** – I do not see IPV as an exclusion criterion in the supplementary file describing study enrolment. It is also not clear for me why did you decide using IPV event as exclusion criterion; could you please elaborate on that?

There were three questions about the history of IPV for each named partner. If any of the three questions were positive, the Partner was excluded from further enrollment because it could potentially increase the risk for women. This was done according to the donor’s guidelines.

**Results:**

A general limitation reg. the analysis is that in this population, even using a few variables, you deal with confounding. Most obvious example: age is correlated with educational level. If any adjustment is not possible in your study sample, I would advise mentioning this point as a study limitation.

Our understanding is that the concept of confounding is pertinent to hypothesis testing, i.e. testing specific associations, particularly when attempting to confirm causal relationships between independent and dependent variables. Our main study question is purely descriptive – to measure the proportion of cases among women that can be linked to PWID. In this context, we believe that confounding is not applicable. We do, test the differences between women with and without a PWID Partner, and between Partners who could be infected via IDU vs not. We agree that in these comparisons the observed associations can be explained by other measures or unmeasured factors /confounders. However, we did not have any hypotheses regarding pre-defined outcomes, and do not attempt to make causal inferences (e.g. ‘having a PWID partner will increase the risk of IPV’), we only report the associations. Therefore, we do not think that making any adjustments, like using multivariable regression to verify the associations is necessary. Nevertheless, we agree that some confounding may be present, and add this as a limitation.

I think it might be of interest to indirectly look at the ‘missing’ group of the partners who did not participate in the study. Was there any age, educational level, or risk behavior difference between the women whose partners agreed to participate, and those whose did not? Was any difference in IPV between these two groups of women? The missing group is likely not missing at random, as one would hypothesize that a person who inject drugs might be more likely to refuse to participate.

Thank you for this valuable insight, we totally agree this was worth investigating. In this version, we conducted this analysis, now presented in Results and Supplementary Table 3. It shows that women who had a Partner in our study were more likely to report sex with PLWH. This may be explained by our recruitment approach, which excluded partners with IPV risks, i.e. women with ‘safe’ Partners were more likely to know about their HIV status and were more likely to recruit them – highlighting the importance of status disclosure. Another difference was the frequency of sex work, which is harder to explain. There were no other differences (importantly, in having sex with PWID), suggesting that the subgroups were largely similar.

Could you please explain why the age group were defined using the cutoff of 26 years? At inclusion, you used a definition of 15-25 years, and five years seem also the more logical time interval for an ‘arbitrary’ age group definition; why 26 years was used at the study enrolment?

Age categories in this version were revised to more standard categories: below 20, 20-24, and 25+. We realize that the standard AGYW definition is 15-24. It is hard to ascertain at this point why women at the age of 25 were included, possibly it was a mistake in the screening formula (≤ instead of <). But we believe this does not affect the validity of main findings, therefore we prefer not to exclude these women (N=31) at this point not to further reduce the sample size.

Line 221-224: “***236 (73%) AGYW indicated at least one sexual partner with negative IPV screening results, and 94 (40%) agreed to refer their partners to the study site. 41 (13%) out of all AGYW reported IPV history.***” – If 73% reported at least one partner with negative IPV screening result, and 13% reported IPV history, even assuming that no IPV history was ever reported in the 73% group, there are still 10% with neither positive nor negative IPV, could you please elaborate on this?

This section was revised, and Supplementary Table S2 was added to clarify this. Each woman could name up to five partners, 0 did not name partners, 181 named one partner, 69 – two partners, etc. For each partner, three questions about IPV and abuse were asked: ‘Did this partner ever beat you or cause physical pain by any means?’, ‘Did this partner ever threaten to harm you?’, ‘Did this partner ever force you during sex to do something causing discomfort for you?’. All three had to be negative to consider the partner ‘safe’. 168 women had one ‘safe’ partner, 52 – two, etc. Together, 267 (83%) had at least one ‘safe’ partner. 41 women responded positively to the first question (‘Did this partner ever beat you or cause physical pain by any means?’) for any partner, which is presented in the tables.

We hope it is now clearer in the manuscript.

For the question on family status, the answers are “single/separated” or “married”. Was cohabitance without marriage reported as “single”?

Unfortunately the questionnaire did not distinguish between single with relationship/cohabitance and totally single. There was a separate category for divorced and widowed, which we now present in the tables.

You could consider excluding the variable “IDU exposure” and “Heterosexual exposure” from Table 1, given that this was an exclusion and inclusion criterion, respectively, therefore by definition you would not have anyone with IDU exposure in your population, and all will be heterosexual.

We agree that heterosexual exposure is obsolete in this study context (it was inherited from the original MoT study). Although we prefer to keep the IDU category as it is describes how self-reported IDU was defined for Partners.

“Time in HIV” care groups look overlapping, whereas the total proportion shows no overlap in participants. Was Year 2 in care included in the “1-2 years” or in the “2-3 years” interval? Same question for the Year 3.

We apologize for confusion. Time in HIV care and time from testing to registration was calculated in days, and then converted to months /years. For clarity, the categories have now been labeled as: <3 months; 3-11.99 months; 12+ months. And <2 years; 2-3.99 years; 4+ years.

The numbers of participants for the three of the four years of registration in care is precisely the same: 85. Was this planned at the selection stage (i.e. you planned to select the same number of participants for each of the study years)? If so, this should be specified in Methods or in Supplementary 2.

The sample was stratified by year of registration, so it was intentional. But we decided to remove this variable from the tables as basically reflects the time in care and does not convey any important information.

Line 228: “***Sex with PWID before HIV diagnosis was reported in 19% of females, and rare or never condom use was reported in 40% of females***” – consider replacing “females” with “women”

Thank you, we agree, the language has been corrected throughout.

Table 2: I suggest deleting “or drugs” and keeping only “Exchange money for sex”, for the same reason I do not recommend keeping “IDU exposure” in Table 1.

Agreed, revisions made accordingly.

Lines 227-231: here in the text, you report the same proportions that are listed in the table. You may consider shortening the text, to minimize duplicate data. I would suggest removing the sentence on sex with MSM and commercial partners.

The section has been revised completely.

Lines 234-236: “***AGYW who reported having IDU sexual partner (IP) had a significantly higher frequency of high-risk heterosexual exposure than AGYW reporting having a non-IDU partner (NIP) (100% vs 59%, p<.0001****)”.* Here, the outcome of interest is used for the group definition, and in this case, there is no variance in one of the groups (IDU partners) and your p will be low even if only one of the participants in the non-IDU partner group does not have a high-risk exposure. You might consider different methodology to compare to a ceiling, or easier, you could simply say “*among AGYW reporting having a non-IDU partner, the frequency of high-risk heterosexual exposure was 59% (95%CI XX-XX)”.*

Thank you for bringing this up, we totally agree that the concept of ‘high-risk heterosexual exposure’ was misleading in this context. We have now revised the analytic approach and removed this definition completely. Instead, we present each risk factor separately (i.e. sex with PLWH, sex with PWID, etc).

I believe the sample size is also too small to assess the difference between groups for exchanging money for sex; sex with MSM; and sex with commercial partner, therefore p-value is meaningless, and I would suggest excluding the sentence describing p-values in these groups.

We agree that the sample sizes are small. In this version we have revised the analytic approach, and now use Fischer’s exact test for subtables with small counts, which we believe is more appropriate. The section has been revised as well.

Lines 236-238: “***In addition, AGYW with IP were more likely to have STI exposure and sex with PLWH than AGYW with NIP (40% vs 24%, p=.011 and 74% vs 55%, p=.005 respectively)”.*** How was “sex with PLWH” defined? Could the woman answer based on her suspicion, or should there be any confirmation of the partner’s HIV status? Additionally, please spell out “PLWH” – it seems for me as it was not spelled before.

The question in the survey was ‘Did you have sex with a man who has HIV?’. It did not require confirmation, so this is indeed based on their knowledge. We agree it may not be 100% accurate, but it does represent a conservative estimate of the prevalence of serodiscordant relationships.

PLWH is now spelled out in tables’ footnotes.

Unfortunately, I could not properly look at Table 3, since a part of it was not visible in the portrait format. Please include this table in landscape format. Additionally, you could shorten the text above the table leaving only the most important findings, to avoid duplicating a large amount of data that is already in the table.

We apologize for the inconvenience. All tables have now been reformatted to fit the page in portrait view.

The language in the section was shortened to mention only the figures that we want to highlight.

Line 241: ”***Rare/never condom use was numerically higher than AGYW with IP***” - a typo: “*was higher IN AGYW with IP*”

Thank you, correction was made.

Lines 257-258: “***and 71% reported consuming alcohol and drugs before sex always or frequently***” – should it rather be “*alcohol or drugs*”?

Yes, it should be ‘or’, correction was made.

In the analysis in partners, some subgroups are very small for the p-value to be able to estimate the difference.

We agree that the sample sizes are small. In this version we have revised the analytic approach, and now use Fischer’s exact test for subtables with small counts, which we believe is more appropriate. It does not solve the problem of type II error, but for those associations that are significant, we may be reasonably sure that there is difference.

In table 5, 0% of partners reported exchanging money or drugs for sex but 33% and 19% reported having sex with a commercial partner. Could you please elaborate on that? Does it mean that the partners in fact replied to the question of “exchanging sex for money or drugs”, i.e. selling, but not buying sex?

Yes, the first question was about selling sex for money or drugs (0% reported that), and the second one was about buying sex.

**Discussion:**

Lines 290-291: “***Our results indicate that AGYW with IDU sexual partners are at higher odds for high-risk heterosexual exposure than non-IDU partners***” – As mentioned in the comments to the Results section, having an IDU sexual partner is one of the definitions of the high-risk behavior (something like if you say “people who were born in Ukraine are more likely to have ever lived in Ukraine than those who were born in another country”).

We agree, this concept was removed entirely.

Lines 291-293: “***In addition, having an IDU sex partner was associated with a higher probability of STI exposure and being more likely to have sex with PLWH***”. – I believe this can be strengthened by the check in the group of women whose partners participated in the study, because in this case, you have a confirmation of the HIV status from the partner or HIV clinic. Have you considered looking into it?

Yes, we agree, the main comparison is now done only within the subgroup of women who had a Partner enrolled in the study (Table 3).

Lines 293-295: “***Similarly, in the study conducted by Deryabina et al. [47] in similar Post-Soviet contextual settings, up to 70% of women reported risky sexual behaviour with their PWID partners***” – The paper does not assess any of the high-risk definitions used in your analysis apart from the sex with PWID (which was an inclusion criteria). For clarity of the comparison, I would advise specifying which parameter was reported by up to 70% women in this study.

Yes, we agree this reference is not appropriate in this context and we removed it. But it is still useful to support the point about non-disclosure of IDU.

Lines 319 – 321: “***Furthermore, the proportion of Partners who in our survey self-reported IDU at the time prior to AGYW HIV diagnosis was strikingly higher than those who have been registered in HIV clinics with IDU mode of HIV transmission***”. This does not seem in line with the Results section:

Lines 262-263: ***More than half (53%) of all Partners were registered in HIV clinic with a heterosexual mode of transmission, and 24% were registered as IDU***.

Lines 265-266: ***During the survey, 19 (29%) of Partners reported IDU as their mode of HIV infection***.

The difference between the 24% registered as IDU and 29% who reported IDU transmission during the survey, does not appear to be very striking.

According to the updated analysis, the magnitude of misclassification was substantial – 45% IDU in the survey vs 31% in the registration system. This is comparable to the previous study from 2013-2015. We have revised this paragraph.

Line 343: “***more lifetime sexual partners and STDs”*** – please spell out “STD” or use STI if this is the same

Thank you, all corrected to STIs, spelled out at first use.

Lines 360-371: I suggest shortening or even deleting this part, considering that HIV disclosure is not investigated in this paper, and its importance is mentioned in the other parts of the manuscript.

Agreed, the section was removed.

Limitations: I would suggest mentioning that, along with the low number of enrolled partners, it is also possible that those who did not respond to the referral, were not missing at random.

Thank you, this limitation was added and discussed.

**Supplementary 1:**

Could you please explain why it has been decided to link one partner to one woman only? Potentially, there is no obstacle for one man to infect multiple women.

Women were able to identify and refer up to 5 partners, and 4 women recruited two partners (see Table S2). But we did not have any means to cross-check whether different women named one man. Partners who took part in the study were unique, i.e. no one has participated as a Partner to different women. We agree that it is theoretically possible that some women in the study could be infected by one man, but we believe this is a very low possibility.
